# Laboratory evaluations of the immediate and sustained effectiveness of lotilaner (Credelio™) against three common species of ticks affecting dogs in Europe

**DOI:** 10.1186/s13071-017-2477-x

**Published:** 2017-11-01

**Authors:** Daniela Cavalleri, Martin Murphy, Regina Lizundia Gorbea, Wolfgang Seewald, Jason Drake, Steve Nanchen

**Affiliations:** 1 Elanco Animal Health, Mattenstrasse 24a, 4058 Basel, Switzerland; 2 Elanco Animal Health, 2500 Innovation Way, Greenfield, Indiana 46140 USA

**Keywords:** Lotilaner, Credelio, Ticks, *Ixodes*, *Rhipicephalus*, *Dermacentor*, Dog

## Abstract

**Background:**

There is a continuing need for novel approaches to tick control in dogs. One such approach lies in the ability of lotilaner **(**Credelio™**)**, an isoxazoline with a rapid onset of action, to provide sustained efficacy against ticks. Two studies were undertaken to confirm lotilaner’s efficacy, at the minimum dose rate of 20 mg/kg, against the three most common tick species in Europe.

**Methods:**

In each of two studies, 16 Beagle dogs, at least 6 months old, were ranked and blocked by tick counts from infestations placed approximately 1 week before treatment. Within blocks, dogs were randomized to receive either lotilaner flavoured chewable tablets at as close as possible to, but not less than the minimum dose rate of 20 mg/kg, or to be sham-treated controls. Study 1 assessed lotilaner efficacy against concurrent infestations with 50 (± 6) *Rhipicephalus sanguineus* and 70 (± 6) *Ixodes ricinus*; Study 2 infestations were with 50 (± 2) *Dermacentor reticulatus.* Infestations were performed on Day -2 with counts on Day 2, 48 (± 2) hours post-treatment. Post-treatment infestations were performed on Days 7, 14, 21, 28 and 35, and ticks were counted 48 (±2) hours post-infestations. Efficacy was determined by the percent reduction in mean live tick counts.

**Results:**

Control group infestations for each tick species were adequate for assessing lotilaner efficacy at all assessment times. On Day 2 no live ticks were found on any lotilaner-treated dog. For subsequent counts, in Study 1 lotilaner was 100% effective in eliminating live *I. ricinus* and *R. sanguineus* on all but two occasions for each tick; on each of those occasions efficacy was sustained at greater than 98.0%. In Study 2, except for a single unattached live tick found on Day 16, efficacy against *D. reticulatus* was 100% at every post-treatment assessment.

**Conclusion:**

The high and sustained efficacy against the three common species of ticks in Europe, *R. sanguineus*, *I. ricinus* and *D. reticulatus*, demonstrates that lotilaner can be a valuable tool in the treatment of canine tick infestations. Lotilaner flavoured chewable tablets were well tolerated and effectiveness was sustained through at least 35 days.

**Electronic supplementary material:**

The online version of this article (doi: 10.1186/s13071-017-2477-x) contains supplementary material, which is available to authorized users. See Additional file 1 for the French translation of the Abstract.

## Background

The report of resistance or tolerance to common topically applied acaricides highlights the need for novel approaches to the treatment of tick infestations on dogs [1]. Fortunately, the emergence of the isoxazolines has begun to address that need. Acting with a unique mode of action, the isoxazolines have been shown to be effective in treating ectoparasitic infestations on dogs [2–4].

The most recent of the isoxazoline family to become available for use in dogs is lotilaner, which is presented as a flavoured, chewable tablet formulation (Credelio™). Early studies showed that oral lotilaner administration to recently fed dogs was followed by rapid absorption, with peak lotilaner blood levels achieved within approximately 2 h, allowing rapid knockdown of both fleas and ticks [5–7]. The lotilaner half-life of 30 days then provides sustained activity through at least a full month following treatment. Safety studies established that the recommended target lotilaner dose rate, 20–43 mg/kg, had a wide safety margin in dogs, and safety has been further confirmed in field studies [8–11].

The efficacy of lotilaner has been demonstrated against *Ixodes scapularis*, *Dermacentor variabilis*, *Rhipicephalus sanguineus* and *Amblyomma americanum* for 1 month, while a laboratory study demonstrated that efficacy against *Ixodes ricinus* was apparent within 4 h after treatment [6, 12]. To provide further confirmation of the efficacy of the minimum recommended dose rate of 20 mg/kg lotilaner and its sustained activity against ticks, two studies were undertaken in dogs challenged with *R. sanguineus* and *I. ricinus* (Study 1), and *Dermacentor reticulatus* (Study 2). These three species have been shown in surveys and field studies to be the three main ticks infesting dogs in Europe [11, 13–16].

## Methods

Studies were completed at two separate laboratories in compliance with the principles of Good Clinical Practices [17]. All study protocols were reviewed and approved by the appropriate Institutional Animal Care and Use Committee (IACUC)/Ethics committee. 

### Animals and housing

To qualify for inclusion in either study, Beagle dogs were required to be at least 6 months old and to be acclimated to the study site and conditions, to be clinically healthy and not pregnant, to have undergone a sufficient washout period following any previous treatment with acaricidal compounds, and to have had a tick attachment rate of at least 25% from an infestation completed approximately 1 week before the day of study treatment. In Study 1, dogs were individually housed throughout the study period, and received regular exercise and social interaction. In Study 2 dogs were pair-housed with dogs from the same treatment group, except when individually housed for 96 h during the initial tick challenge period (Day -2 to Day 2), and 48 (± 2) hours subsequent to challenges performed after treatment.

During the study, room temperatures remained within the range of 17.6 °C and 29.5 °C, and relative humidity within the range 20.0–80.0%. Each facility provided a photoperiod of 12 h light:12 h darkness using overhead lighting. All dogs had access to water ad libitum and were fed a commercially available, high quality, complete canine diet according to each facility’s standard procedure.

### Tick infestations and counts

For inclusion in the study and randomization, initial tick infestations were completed on Day -5 or -6, and counts completed 48 h later. For efficacy assessments, tick infestations were completed on Day -2, and ticks were counted and removed on Day 2, 48 (± 2) hours post treatment. Post-treatment infestations were then performed on Days 7, 14, 21, 28 and 35, and ticks were counted and removed on Days 9, 16, 23, 30 and 37, at 48 (± 2) hours post-infestation. For tick infestations dogs were sedated and infested while in individual cages. After infestations, dogs remained in those cages for up to 1 h in Study 1, and up to 6 h in Study 2. The dogs were returned to their individual crates when they had fully recovered from the effects of sedation.

Study 1 assessed the efficacy of lotilaner using infestations of approximately 50 (± 6) *R. sanguineus,* and concurrent infestations with approximately 70 (± 6) *I. ricinus*; Study 2 assessed efficacy against infestations of 50 (± 2) *D. reticulatus.* For all infestations adult, unfed ticks were used, with a ratio of female:male ticks of approximately 1:1. All study ticks were obtained from laboratory-maintained colonies which had been initiated with European field isolates and that were refreshed at regular intervals with additional field-caught ticks from different European areas.

The tick-counting procedure consisted of thorough examination of all body areas by careful palpation to locate and count the attached/free ticks, followed by combing to remove all ticks from the dog. Ticks were considered alive if legs reacted to a tactile stimulus or exhaled CO_2_ and were considered dead if they did not. Ticks were considered attached if the tick’s mouth part was firmly attached to the dog’s skin and were considered free if it was not. To further sub-categorize ticks as unengorged or engorged, attached ticks were placed between sheets of white paper towel and squeezed. If blood (or dark colored liquid) was visible on the paper, the tick was recorded as engorged.

### Randomization and treatments

Dogs were ranked in descending order of tick counts from the infestations on Days -5 or -6. In each study, the 16 dogs that had the highest tick counts (live attached ticks) and at least a 25% attachment rate were enrolled in the study, provided they met all other inclusion criteria and no exclusion criteria. Dogs were rank-ordered from highest to lowest tick counts and randomly allocated within blocks of two to treatment groups.

Dogs in one group received a single dose of lotilaner flavoured chewable tablets, administered orally at as close as possible to, but not less than the minimum dose rate of 20 mg/kg. Dogs in the other group were sham-treated negative controls. Sham treatment consisted of dogs being brought to the examination room and placed on the table as for the treated animals, or by the treatment administrator entering the pen and opening and massaging the dog’s mouth.

Except for one dog, all dogs had consumed at least one third of the daily ration within approximately 30 min prior to dosing. On Day 0, treatments were administered directly into each dog’s mouth to ensure the targeted dose was delivered. Actual lotilaner dose rates ranged from 20.4 to 22.7 mg/kg.

### Efficacy assessments

In both studies efficacy was determined by the percent reduction in counts of live ticks on lotilaner-treated dogs compared to the counts in the control group. Efficacies based on geometric and arithmetic means were calculated according to Abbott’s formula as follows:

Efficacy (%) = 100 × (MC – MT) / MC, where MC is the mean number of live ticks on dogs in the untreated control group, and MT is the mean number of live ticks on dogs in the treated group.

In compliance with the previous CVMP (Committee for Medicinal Products for Veterinary Use) Guideline for the testing and evaluation of the efficacy of antiparasitic substances for the treatment and prevention of tick and flea infestation in dogs and cats, similar calculations were performed to include dead engorged ticks in the counts [18]. The attachment rate of *I. ricinus* male ticks was not considered as only females of this species attached to a dog.

Since the calculation of the geometric mean involved taking the logarithm of the parasite count of each dog, when any of the parasite counts were equal to zero a one (1) was added to the count for every dog. In these cases one (1) was subtracted from the resultant calculated geometric mean prior to calculating percent effectiveness.

Lotilaner was considered efficacious against the tested tick species at a given time point if the following criteria were met: (i) Dogs in the negative control group had an average attachment rate of at least 25%; (ii) There was a statistically significant difference (*P* < 0.05) between the treated group and the untreated control group; (iii) The treated group had a calculated efficacy of ≥ 90%.

### Translation

French translation of the Abstract is available in Additional file 1.

## Results

Control group infestations for each tested tick species met the criterion of adequacy to assess lotilaner efficacy at all assessment time-points. In Study 1, there were at least 11 live attached female *I. ricinus* on each of the eight control dogs, and at least nine live attached *R. sanguineus* at each time-point, and in Study 2, there were at least 15 *D. reticulatus* ticks on each of the control dogs at each assessment.

**Fig. 1:**
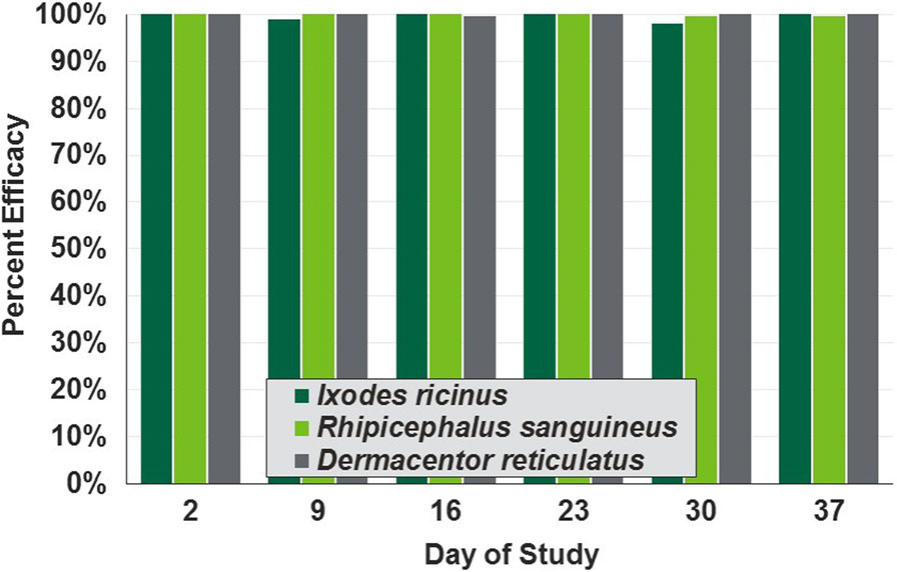
Percent reductions in geometric mean live counts of *Ixodes ricinus*, *Rhipicephalus sanguineus* and *Dermacentor reticulatus* ticks in lotilaner-treated dogs. Geometric mean counts of each tick were significantly lower in lotilaner-treated dogs than in control dogs at each assessment point (*P* < 0.0001)

Against the three tick species tested, on Day 2 (48 h post-treatment) no live ticks were found on any lotilaner-treated dog (Tables 1 and 2, Fig. 1). The high efficacy against ticks was sustained in all studies throughout the post-treatment assessment periods. For counts completed on and after Day 9, in Study 1 lotilaner was 100% effective in eliminating live *I. ricinus* and *R. sanguineus* on all but two occasions for each tick, and on these occasions efficacy was at least 98.0%. In Study 2, efficacy against *D. reticulatus* was 100% at all but one of the post-treatment assessments. At that one assessment, on Day 16, a single live, free tick was found on one dog.

**Table 1:** Geometric (arithmetic) mean counts of live *Ixodes ricinus* and *Rhipicephalus sanguineus* ticks in Study 1

Tick species	Day	Untreated control		Lotilaner			
		Mean	Range	Mean	Range	Efficacy (%)	Comparison
*Ixodes ricinus*	2	18.3 (18.6)	14–26	0.0 (0.0)	0–0	100 (100)	*t*_(14)_ = 46.6, *P* < 0.0001
	9	24.5 (24.8)	20–28	0.3 (0.4)	0–1	98.8 (98.5)	*t*_(14)_ = 21.9, *P* < 0.0001
	16	22.9 (23.6)	13–32	0.0 (0.0)	0–0	100 (100)	*t*_(14)_ = 32.6, *P* < 0.0001
	23	23.6 (25.1)	11–33	0.0 (0.0)	0–0	100 (100)	*t*_(14)_ = 23.0, *P* < 0.0001
	30	24.0 (24.6)	16–35	0.5 (0.8)	0–3	98.0 (97.0)	*t*_(14)_ = 12.7, *P* < 0.0001
	37	24.2 (25.1)	12–35	0.0 (0.0)	0–0	100 (100)	*t*_(14)_ = 29.4, *P* < 0.0001
*Rhipicephalus sanguineus*	2	25.0 (26.8)	9–40	0.0 (0.0)	0–0	100 (100)	*t*_(14)_ = 21.4, *P* < 0.0001
	9	29.7 (30.5)	18–39	0.0 (0.0)	0–0	100 (100)	*t*_(14)_ = 37.6, *P* < 0.0001
	16	27.4 (28.6)	15–37	0.0 (0.0)	0–0	100 (100)	*t*_(14)_ = 28.7, *P* < 0.0001
	23	31.4 (32.3)	21–48	0.0 (0.0)	0–0	100 (100)	*t*_(14)_ = 40.8, *P* < 0.0001
	30	31.8 (32.6)	19–43	0.1 (0.1)	0–1	99.6 (99.7)	*t*_(14)_ = 27.6, *P* < 0.0001
	37	29.8 (30.8)	17v41	0.1 (0.1)	0–1	99.6 (99.7)	*t*_(14)_ = 25.4, *P* < 0.0001

**Table 2:** Geometric (arithmetic) mean counts of live *Dermacentor reticulatus* ticks in Study 2

Tick species	Day	Untreated control		Lotilaner			Comparison
		Mean	Range	Mean	Range	Efficacy (%)	
*Dermacentor reticulatus*	2	45.6 (45.8)	40–52	0.0 (0.0)	0–0	100 (100)	*t*_(14)_ = 116.7, *P* < 0.0001
	9	34.6 (35.0)	26–42	0.0 (0.0)	0–0	100 (100)	*t*_(14)_ = 60.7, *P* < 0.0001
	16	37.3 (38.0)	23–48	0.1 (0.1)^a^	0–1	99.8 (99.7)	*t*_(14)_ = 31.1, *P* < 0.0001
	23	32.7 (33.4)	19–42	0.0 (0.0)	0–0	100 (100)	*t*_(14)_ = 42.8, *P* < 0.0001
	30	29.8 (31.6)	16–48	0.0 (0.0)	0–0	100 (100)	*t*_(14)_ = 26.0, *P* < 0.0001
	37	28.1 (29.3)	15–44	0.0 (0.0)	0–0	100 (100)	*t*_(14)_ = 30.6, *P* < 0.0001

^a^A single live free tick was found on one dog in the treated group

Assessments of lotilaner efficacy also included reductions, relative to controls, of live ticks plus dead engorged ticks. Two days after treatment of tick-infested dogs, in Study 1 there were only two dead attached and engorged *I. ricinus* on each of four dogs in the eight-dog treatment group, and two dead attached engorged *R. sanguineus* on a single dog in the eight-dog treatment group (this dog did not have any attached *I. ricinus* ticks). No other dead attached engorged *R. sanguineus* were found on lotilaner-treated dogs. In Study 2 there were no dead engorged *D. reticulatus* (*i.e,* there were no ticks found on any of the eight lotilaner-treated dogs). Overall, for Days 9 through 37, when dead engorged ticks were included in counts, for *I. ricinus* lotilaner efficacy remained at greater than 95%, except on Day 30 when the arithmetic mean reduction, relative to control dogs, was 93.4% (efficacy at this point based on geometric mean was 95.1%). Against *R. sanguineus* and *D. reticulatus*, lotilaner efficacy based on geometric and arithmetic mean reductions in live plus dead attached engorged ticks remained at greater than 99% throughout the study (Tables 3, 4).

**Table 3:** Geometric (arithmetic) mean counts of live *Ixodes ricinus* and *Rhipicephalus sanguineus* ticks and dead, attached, engorged ticks in Study 1

Tick species	Day	Untreated control		Lotilaner			Comparison
		Mean	Range	Mean	Range	Efficacy (%)	
*Ixodes ricinus*	2	18.3 (18.6)	14–26	0.7 (1.0)	0–2^a^	96.0 (94.6)	*t*_(14)_ = 11.1, *P* < 0.0001
	9	24.5 (24.8)	20–28	0.6 (0.8)	0–2	97.7 (97.0)	*t*_(14)_ = 15.1, *P* < 0.0001
	16	22.9 (23.6)	13–32	0.5 (0.8)	0–3	97.9 (96.8)	*t*_(14)_ = 12.3, *P* < 0.0001
	23	23.6 (25.1)	11–33	0.6 (0.9)	0–2	97.3 (96.5)	*t*_(14)_ = 11.3, *P* < 0.0001
	30	24.0 (24.6)	16–35	1.2 (1.6)	0–4	95.1 (93.4)	*t*_(14)_ = 9.4, *P* < 0.0001
	37	24.2 (25.1)	12–35	0.7 (1.0)	0–3	97.1 (96.0)	*t*_(14)_ = 11.2, *P* < 0.0001
*Rhipicephalus sanguineus*	2	25.0 (26.8)	9–40	0.1 (0.3)	0–2^b^	99.4 (99.1)	*t*_(14)_ = 15.2, *P* < 0.0001
	9	29.7 (30.5)	18–39	0.0 (0.0)	0–0	100 (100)	*t*_(14)_ = 37.6, *P* < 0.0001
	16	27.4 (28.6)	15–37	0.0 (0.0)	0–0	100 (100)	*t*_(14)_ = 28.7, *P* < 0.0001
	23	31.4 (32.3)	21–48	0.0 (0.0)	0–0	100 (100)	*t*_(14)_ = 40.8, *P* < 0.0001
	30	31.8 (32.6)	19–43	0.1 (0.1)	0–1	99.7 (99.6)	*t*_(14)_ = 27.6, *P* < 0.0001
	37	29.8 (30.8)	17–41	0.1 (0.1)	0–1	99.7 (99.6)	*t*_(14)_ = 25.4, *P* < 0.0001

^a^48 h post-treatment live or dead engorged ticks were found on a single; this dog had two dead, engorged *R. sanguinueus*

^b^48 h post-treatment four dogs were infested, each with two dead engorged *I ricinus*

**Table 4:** Geometric (arithmetic) mean counts of live *Dermacentor reticulatus* ticks and dead, attached, engorged ticks in Study 2

Tick species	Day	Untreated control		Lotilaner			Comparison
		Mean	Range	Mean	Range	Efficacy (%)	
*Dermacentor reticulatus*	2	45.6 (45.8)	40–52	0.4 (0.6)	0–2^a^	99.0 (98.6)	*t*_(14)_ = 18.8, *P* < 0.0001
	9	34.6 (35.0)	26–42	0.0 (0.0)	0–0	100 (100)	*t*_(14)_ = 60.7, *P* < 0.0001
	16	37.3 (38.0)	23–48	0.2 (0.3)	0–2^b^	99.6 (99.3)	*t*_(14)_ = 22.5, *P* < 0.0001
	23	32.7 (33.4)	19–42	0.0 (0.0)	0–0	100 (100)	*t*_(14)_ = 42.8, *P* < 0.0001
	30	29.8 (31.6)	16–48	0.0 (0.0)	0–0	100 (100)	*t*_(14)_ = 26.0, *P* < 0.0001
	37	28.1 (29.3)	15–44	0.0 (0.0)	0–0	100 (100)	*t*_(14)_ = 30.6, *P* < 0.0001

^a^On Day 2, 48 hours post-treatment, ticks on three dogs, all were dead, attached and engorged

^b^On Day 16, a single lotilaner dog was infested. This dog had one live free tick and one dead attached/engorged tick

In Study 1, mucoid blood was observed intermittently in the faeces of a dog in the untreated control group during inclusion/exclusion clinical examinations. A faecal examination confirmed *Giardia* infection as the cause; one dog in the treated group also presented with blood in the faeces. A faecal flotation test was negative, no diagnosis was made and the dog recovered without treatment. There were no other adverse clinical signs observed in lotilaner-treated dogs. In Study 2, transient increased salivation was observed in one lotilaner-treated dog which was attributed to excitement. There were 10 and 6 observations of diarrhea/loose stool in the treated and control groups, respectively, and four recordings of vomition in lotilaner-treated dogs. As dogs were housed by pairs, the clinical sign was automatically attributed to both dogs present in the cage. These were single events (dogs vomited once), and appeared 21 days after treatment administration. No concomitant treatment was needed, and all gastrointestinal signs resolved uneventfully without treatment. Skin crusts attributable to the tick infestation model were observed in dogs in both groups, but more frequently in control dogs.

## Discussion

The results of this study align with other reports of the effectiveness of orally administered lotilaner against a range of tick species that infest dogs. In other studies, the sustained efficacy of lotilaner has been demonstrated through at least 1 month for *D. variabilis*, *I. scapularis*, *R. sanguineus* and *A. americanum*, indicating that there is no tapering in efficacy at the end of the recommended monthly treatment interval [12].

The rapid onset of lotilaner’s activity against ticks has been previously demonstrated [6]. Lending further support to the knockdown effect of existing infestations is the finding that there were no, or very low numbers of ticks on lotilaner-treated dogs, while many were present on the control dogs. This is relevant in reducing the risk of client perceptions of product failure if ticks, even if dead, are observed on a dog in the days immediately following treatment.

While speed of action of an acaricide is important, both in killing infestations present when treatment is administered, and in quickly killing newly infesting ticks in the post-treatment period, a requirement for registration is to demonstrate effectiveness at 48 h post-treatment and 48 h following weekly post-treatment challenges for at least the duration of the sought indication. In this regard, when the results of the studies reported here are taken with the results of the other investigations of activity against ticks, lotilaner meets and often exceeds the efficacy reported both for orally-administered and topically-applied monthly tick-control products, consistently providing sustained effectiveness throughout the month following treatment.

Against *R. sanguineus*, in the study reported here and in a report of three earlier studies, lotilaner consistently demonstrated efficacy of 99 to 100% from Days 2 through 35 [12]. For afoxolaner and sarolaner assessed through 35 days post-treatment, reported efficacy against *R. sanguineus* has ranged from 95.7 through 100%, and from 99.5 to 100%, respectively [19, 20].

For three topically applied products - combinations of dinotefuran/pyriproxyfen/ permethrin; fipronil/(S)-methoprene; and imidacloprid/permethrin - efficacy against *R. sanguineus* ranged from 95.0 to 100% on Days 9, 16 and 23. The Day 30 finding of 98.5, 92.8 and 91.8% efficacy for dinotefuran/pyriproxyfen/permethrin, imidacloprid/permethrin, and fipronil/(S)-methoprene, respectively, is suggestive of a tapering in efficacy at the end of the month following treatment [21]. Such tapering was also reported from a separate study in which efficacy of the dinotefuran/pyriproxyfen/permethrin product was 93.5 to 100% against infestations applied weekly from seven to 28 days after treatment, declining to 90.4% on Day 35 [22]. This tapering effect was not as clear in a study of a topically applied imidacloprid/permethrin combination, but for that product the 48-h post-challenge efficacy against *R. sanguineus* was only 92.0 and 95.9% at 21 and 28 days post-treatment, respectively [23].

The studies with these topically applied products also indicate that their onset of activity is slower than that of lotilaner. While lotilaner efficacy was 100% at 48 h post-treatment, for the above-mentioned topical products efficacy against *R. sanguineus* ranged from 23.9 to 75.5% at 48 h post-treatment [21–23]. Another comparative study demonstrated that when administered orally, isoxazolines provided a more effective approach to the treatment of existing tick infestations than did a topical combination formulation of imidacloprid and permethrin [24]. This slow onset of activity of topical products has been attributed to the delay in the distribution of the active chemicals across the treated dog’s body surface [21].

Against *I. ricinus*, the investigation reported here found lotilaner efficacy to be at least 98.9% at all assessments, with complete (100%) removal of infestations on the final study challenge on Day 35 (when dead, engorged ticks were included in the count, efficacy was at least 95% throughout the study, and was 97.1% on Day 35). Afoxolaner and sarolaner have also shown high efficacy against this tick [20, 25]. Topical combinations of imidacloprid/permethrin and permethrin/fipronil and were each reported to have efficacy of 98 to 100% following weekly challenges up to 3 weeks post-treatment, for permethrin/fipronil declining to 93% on Day 30 [23, 26].

Against *D. reticulatus*, except for a single live, free tick found on Day 16, lotilaner efficacy was 100% at every post-treatment assessment through Day 35. Against this tick species, afoxolaner and sarolaner have demonstrated a high level of efficacy, although in one study efficacy of the former product had declined to 96.4% at Day 30 [20, 25].

Other reports of acaricidal effectiveness of different products for use in or on dogs have based efficacy calculations on live tick counts only. In compliance with the CVMP guideline which was current at the time these studies were conducted, efficacy assessments in the studies we report here were also based on live tick counts plus dead, attached engorged tick counts [18]. This is the first report in which isoxazoline efficacy against ticks has been reported in this manner. A rationale for this approach is that a tick which is attached and has engorged can have the potential to transmit disease-producing pathogens. Even using this more stringent approach to assessing tick kill, lotilaner efficacy was sustained at 95% or greater throughout the study with no tapering effect at the end of 1 month post-treatment.

## Conclusion

The high efficacy against three common species of ticks in Europe, *Rhipicephalus sanguineus*, *Ixodes ricinus* and *Dermacentor reticulatus*, demonstrates the potential for lotilaner to be a valuable tool in the treatment of tick infestations of dogs. Lotilaner flavoured chewable tablets were well tolerated and efficacy was sustained through at least 35 days.

## Additional file

Additional file 1: French translation of the Abstract. (PDF 36 kb)

## Abbreviations

AN(C)OVA: Analysis of (co)variance
CVMP: Committee for Medicinal Products for Veterinary Use
IACUC: Institutional Animal Care and Use Committee
