# Supplementary material for: Laboratory evaluations of the immediate and sustained effectiveness of lotilaner (Credelio™) against three common species of ticks affecting dogs in Europe
Source: Parasit Vectors. 2017 Nov 1;10:527. doi: 10.1186/s13071-017-2477-x (PMC5664927; doi:10.1186/s13071-017-2477-x)
Supplement: Additional file 1: — French translation of the Abstract. (PDF 36 kb) [file 13071_2017_2477_MOESM1_ESM.pdf]

Évaluations en laboratoire de l'efficacité immédiate et résiduelle du lotilaner (Credelio™) contre trois espèces de tiques infestant fréquemment le chien en Europe

Daniela Cavalleri[1], Martin Murphy[1], Regina Lizundia Gorbea[1], Wolfgang Seewald[1], Jason Drake[2]* et Steve Nanchen[1]

[1]Elanco Santé animale, Schwarzwaldallee 215, CH-4058 Bâle, WRO-1032.2.58, Suisse
[2]Elanco Santé animale, 2500 Innovation Way, Greenfield, IN 46140, États-Unis

*Correspondance : drake_jon_j@elanco.com

Daniela Cavalleri Adresse électronique : cavalleri_daniela_a@elanco.com
Martin Murphy Adresse électronique : murphy_martin_gerard@elanco.com
Regina Lizundia Gorbea Adresse électronique : regina_lizundia@elanco.com
Wolfgang Seewald Adresse électronique : seewald_wolfgang@elanco.com
Jason Drake Adresse électronique : drake_jon_j@elanco.com
Steve Nanchen Adresse électronique : nanchen_steve@elanco.com

Résumé

Contexte : le contrôle des tiques constitue un enjeu permanent qui nécessite sans cesse de nouvelles approches. L'une d'entre elles repose sur la capacité du lotilaner (Credelio™), un agent d'action rapide de la classe des isoxazolines, à exercer un effet rémanent contre les tiques. Deux études ont été conduites afin de confirmer l'efficacité du lotilaner à la dose minimale de 20 mg/kg contre les trois espèces de tiques les plus fréquentes en Europe.

Méthodes : lors de chacune des deux études, 16 chiens de race beagle, âgés d'au moins six mois, ont été classés et regroupés dans des blocs en fonction du nombre de tiques récoltées après leur infestation environ une semaine avant le traitement. Au sein des blocs, les chiens ont été randomisés pour recevoir des comprimés à croquer aromatisés à base de lotilaner à une dose aussi proche que possible, mais au moins égale au minimum de 20 mg/kg ou dans un groupe de témoins recevant un traitement fictif. L'étude 1 visait à évaluer l'efficacité du lotilaner contre l'infestation simultanée par

50 (± 6) *Rhipicephalus sanguineus* et 70 (± 6) *Ixodes ricinus* ; lors de l'étude 2 les chiens étaient infestés par 50 (± 2) *Dermacentor reticulatus.* Les chiens ont été infestés à J-2 et les tiques dénombrées à J2, 48 heures (± 2) après le traitement. D'autres infestations ont été réalisées après le traitement à J7, J14, J21, J28 et J35, les tiques étant dénombrées 48 (± 2) heures après chaque infestation. L'efficacité était déterminée par la diminution, en pourcentage, du nombre moyen de tiques vivantes.

Résultats : le niveau d'infestation du groupe témoin étant suffisant pour chaque espèce de tique, l'efficacité du lotilaner a pu être évaluée conformément au calendrier de l'étude. A J2, aucune tique vivante n'a été isolée sur aucun chien traité par le lotilaner. Lors des comptages suivants de l'étude 1, l'efficacité du lotilaner a été de 100 % pour éliminer les tiques vivantes *I. ricinus* et *R. sanguineus,* à deux exceptions près pour chaque espèce ; pour chacune de ces exceptions, l'efficacité a été maintenue à un taux supérieur à 98,0 %. Dans l'étude 2, à l'exception d'une seule tique vivante, non fixée isolée à J16, l'efficacité contre *D. reticulatus* a atteint 100 % lors de chacune des évaluations réalisées après le traitement.

Conclusion : la grande efficacité, prolongée, contre les trois espèces de tiques fréquemment rencontrées en Europe, *R. sanguineus, I. ricinus* et *D. reticulatus,* montre que le lotilaner peut constituer un outil particulièrement intéressant dans le traitement des infestations du chien par les tiques. Les comprimés à croquer aromatisés à base de lotilaner ont été bien tolérés et leur efficacité a persisté pendant au moins 35 jours.
